# Prevalence,awareness and factors associated with hypertension in North West Tanzania

**DOI:** 10.1080/16549716.2017.1321279

**Published:** 2017-06-09

**Authors:** Neema R. Mosha, Michael Mahande, Adinan Juma, Innocent Mboya, Rob Peck, Mark Urassa, Denna Michael, Jim Todd

**Affiliations:** ^a^ Mwanza Intervention Trials Unit, National Institute for Medical Research, Mwanza, Tanzania; ^b^ Kilimanjaro Christian Medical Centre, Ministry of Health, Moshi, Tanzania; ^c^ Social Welfare, Moshi and Kilimanjaro Christian Medical University College, Moshi, Tanzania; ^d^ Kilimanjaro Christian Medical University College, Moshi, Tanzania; ^e^ Department of Medicine, Weill Bugando School of Medicine, Mwanza, Tanzania; ^f^ Center for Global Health, Weill Cornell Medical College, New York, NY, USA; ^g^ National Institute for Medical Research, Mwanza, Tanzania; ^h^ Department of Population Health, London School of Hygiene and Tropical Medicine, London, UK

**Keywords:** Hypertension, prevalence, tanzania, community-based, adult, risk factors

## Abstract

**Background**: Hypertension is a public health problem, and yet few people are aware of it and even fewer access effective treatment. With the ongoing demographic transition in many parts of Sub-Saharan Africa, people are changing from rural, manual work to urban lifestyles, hence the risk of hypertension increases.

**Objective**: This study aimed at determining the prevalence, awareness and risk factors associated with hypertension in North West Tanzania.

**Design**: A community-based cross-sectional study was conducted among adults in Magu District in 2013. Information on socio-demographic, economic and lifestyle characteristics, medical conditions, and risk factors for hypertension were collected according to the WHO Steps survey tool. Measurements of blood pressure, blood sugar, pulse rate, and anthropometry were taken. Multivariate logistic regression was used to estimate the odds ratios (OR) and 95% confidence intervals (95% CI) for factors associated with hypertension (Blood pressure ≥140/90mm/Hg). Frequencies and percentages were used to determine the awareness, and treatment among hypertensive participants.

**Results**: Among 9678 participants, the prevalence of hypertension was 8.0% and pre-hypertension 36.2%. There was a higher prevalence of hypertension at older ages, among females (8.2%) compared to males (7.7%), and among urban dwellers (10.1%) compared to rural residents (6.8%). Overweight, obese, and diabetic individuals had a higher risk of hypertension while HIV positive participants had a lower risk of hypertension (OR = 0.56; 95% CI 0.39 – 0.79). Among participants with hypertension, awareness was less than 10%.

**Conclusion**: By integrating blood pressure screening into our long-standing community HIV screening program, we were able to identify many previously undiagnosed cases of hypertension and pre-hypertension. Age, residence, overweight and obesity were the major associated factors for hypertension. Awareness and treatment rates are very low indicating the need for programs to improve awareness, and treatment of hypertension.

## Background

Hypertension is a major risk factor for cardiovascular disease (CVD) [1] and is of increasing public health importance in low- and middle-income countries [2]. Worldwide, there are almost one billion people living with hypertension [3] while different estimates have been found in Sub-Saharan Africa (SSA) [4]. Recent literature shows the problem to be higher among individuals over 40 years of age, Africans, and those with a family history of hypertension or diabetes [5–7]. There are number of modifiable, lifestyle factors associated with hypertension including obesity, overweight, cigarette smoking, alcohol, and low physical activity [8–10]. And there are some reports of an association between hypertension and HIV although this may be due to the use of antiretroviral drugs which may cause increased blood pressure [11]

In countries undergoing the transition from rural, agricultural work to more globalized urban lifestyles the prevalence of hypertension is likely to increase [5,10]. In Tanzania a number of studies have shown a high population prevalence of hypertension ranging from 19% in rural areas to 35% in urban areas, with the highest prevalence of 70% found among individuals aged 70 years and above [12]. A review of the prevalence of hypertension across developing countries showed that the prevalence in the Tanzanian population is higher than in many other countries, and will clearly place a substantial strain on the health services [13].

Despite the increase in hypertension cases in low-income countries, awareness of the problem is low, both among health care workers and in the population at large [14]. Awareness of hypertension is an important factor for early identification and management, but it is poor in many low-income countries [15,16]. Community-based blood pressure screening may be an important way to raise awareness, and refer those with hypertension to receive health services, and this could be integrated with screening for other diseases and conditions such as HIV. Effective treatment and control of hypertension are important for reducing the risk of stroke, heart attack, and other cardiovascular problems, and ultimately, in reducing mortality [17].

The main objective of this study was to determine the prevalence of hypertension, and the associated factors, after blood pressure screening was integrated into a community-based HIV screening program. This population is transitioning from a rural society, to the suburban environment of Mwanza City, thus these results will provide an important baseline for future studies on the prevalence of hypertension in this cohort.

## Materials and methods

### Study design and setting

The Magu Health and Demographic Sentinel Surveillance (HDSS) has been under continuous enumeration since 1994 in six villages about 20 km east of Mwanza city in North West Tanzania [13,14]. In 2012, the villages had a total population of 32 000, with 50% of the population aged 15 years or less, and an average household size of 7.2 individuals. From 7 December 2012 to 31 July 2013 an epidemiological health survey and HIV screening program was carried out recruiting all eligible, adult residents aged 15 years or more, from the HDSS [18]. A total of 9676 adult participants were included in the study which represents almost two third of the adult population included in the Demographic Health Survey. The sample size was estimated that we would have around 400 participants for each sex in each of the 10-year age groups. This would enable us to estimate the prevalence of hypertension with a precision of ±3%.

## Data collection methods and tools

Questionnaires were developed, piloted and translated into Kiswahili (the national language) and KiSukuma (the local language), to collect socio-demographic, economic, behavioural and health information. In the questionnaire, questions from the WHO Steps Survey tool were used for personal and family history of diabetes and hypertension awareness, whether participants have ever had their blood pressure measured before, and if they had received treatment for hypertension [19]. Weight (in kilograms), height (in centimetres) and waist circumference (in centimetres) were measured. Three blood pressure measurements were taken by six trained research nurses using an aneroid-sphygmomanometer, after five minutes of rest, while the participant was quietly waiting for clinical and laboratory services, and the mean of the two last blood pressure measurement was used for analysis. A finger prick dried blood spot was taken from every participant and later anonymously tested for HIV in a central laboratory in Mwanza according to agreed protocols [20]. From the same finger prick, a blood sugar test was performed to screen for the presence of diabetes. The survey procedures were overseen by two field team leaders who supervised the data collection, the clinical services (including the blood pressure readings by the trained nurses), and the laboratory test procedures. Participants who were informed of their HIV positive test result, or high blood pressure, were referred to the government health centre for further investigation, management, and treatment.

## Definition of variables

Hypertension was defined as an average systolic blood pressure (SBP) above or equal to 140 mmHg or diastolic blood pressure (DBP) above or equal to 90mmHg, and pre-hypertension as SBP between 120–139.9 mmHg or DBP between 80–89.9 mmHg [21]. Awareness of hypertension was taken as a positive response to the question ‘Have you ever been told by a doctor or other health care worker that you have raised blood pressure or hypertension?’; and previous treatment through the positive response to the question ‘Have you ever received any drugs (medications) for high blood pressure prescribed by a doctor or other health care worker?’. Aggregated body mass index (BMI) was calculated from the measured weight and height, and categorized as underweight (BMI <18.5), normal (18.5–24.9), overweight (25–29.9), and obese (BMI≥30).Waist circumference was categorized into tertiles for analysis. Raised blood sugar was defined with readings of ≥200 mg/dL and normal blood sugar readings of ≤199.9 mg/dL [22]. Cigarette smoking was categorized into a binary variable as current smokers, and those who are not current smokers (previous or never). Alcohol consumption was categorized into three as: Never, Occasional (one alcoholic drink per week) and Frequent (more than one drink per week). Physical activity was also categorized into three groups as: None, Moderate/Light (carrying light loads, weeding, laundry, herding animals, or using a bicycle or canoe) and Heavy (carrying heavy loads, digging, hoeing, pounding, or chopping).

## Data analysis

Data was cleaned, processed, and transferred from CSPro to STATA Version 12.0 (Stata Corp, USA) for analysis.The proportion of participants with hypertension and pre-hypertension were determined for all demographic and socio-economic characteristics and compared using a chi-squared test. The age standardized proportion with hypertension and pre-hypertension were calculated using the WHO standard population for low- and middle-income countries (LMIC) [23]. The mean anthropometric measurements and blood pressure were compared across participant characteristics using the T-test. Bivariate logistic regression was used to determine the unadjusted odds Ratios (OR) and 95% confidence intervals (CI) for the association between the characteristics and hypertension. Multivariate logistic regression models were fitted to adjust the association between hypertension and risk factors for potential confounders.

## Ethical consideration

Written consent was obtained from all participants. Ethical approval for the surveys was obtained from the Tanzanian National Medical Research Coordinating Committee under the National Institute for Medical Research (Reference MR/53/100/22) and from London School of Hygiene and Tropical Medicine(Reference 7191). Kilimanjaro Christian Medical University college ethics committee gave approval for the use of these data for an MSc project by NM, and permission to use the data was obtained from the TAZAMA Project, and Magu District Medical Officer.

## Results

### Demographic and study characteristics

A total of 9742 individuals were enrolled in the study, 64 (0.6%) of participants were excluded with missing blood pressure data, so 9678 individuals were analyzed. Of these, there were 6277 (65%) females enrolled with a median age of 29 years (interquartile range of 20 to 45 years).

Male participants were significantly taller and heavier than females, however female participants had higher BMI, larger waist circumference, and higher blood sugar result compared with males P-Values < 0.001 (Table 1). There was no statistical significant difference between male and female participants with regard to mean systolic and diastolic blood pressure, or mean pulse rate. Overall in this population, there were 3507 people with pre-hypertension giving a prevalence of 36.2%, and 773 people with hypertension giving a prevalence of 8.0%. The proportion of males with pre-hypertension was higher than females (37.6% vs. 35.4%, P-value 0.029), but the proportion of males with hypertension was lower than that of females (7.7% vs. 8.2%, p-value < 0.05). Between the ages of 15 to 55 years, the proportion with pre-hypertension and the proportion with hypertension increases with the age of the participants (Table 2). After the age of 55 years, the proportion with pre-hypertension decreases although the proportion with hypertension increases to 30.8% in those aged 65 years or more.

**Table 1.** Characteristics of resident’s in Magu HDSS by sex (n = 9678).

		Sex n (%)		
Characteristics	Total	Male n = 3401 (35.1%)	Female n = 6277 (64.9%)	p-value
**Age (years) median (IQR)**	29 (20–45)	26 (18–44)	31 (21–46)	<0.001**
15–24	3763 (38.9)	1585 (46.7)	2178 (35.2)	<0.001
25–34	2018 (21.2)	551 (16.2)	1467 (23.7)	
35–44	1439 (15.0)	434 (12.8)	1005 (16.2)	
45–54	1043 (10.8)	345 (10.2)	698 (11.2)	
55–65	618 (6.5)	228 (6.7)	390 (6.3)	
65+	704 (7.3)	253 (7.4)	451 (7.3)	
**Education level**				
None	2724 (28.2)	608 (17.9)	2116 (33.7)	<0.001
Not completed primary	658 (6.8)	304 (8.9)	354 (5.6)	
Completed primary	4798 (49.6)	1714 (50.5)	3084 (49.1)	
Secondary	1242 (12.8)	604 (18.0)	638 (10.2)	
Tertiary/Other	249 (2.6)	166 (4.9)	83 (1.3)	
**Marital status**				
Single/Never married	3200 (33.1)	1691 (49.7)	1509 (24.1)	<0.001
Married/Cohabiting	4991 (51.6)	1533 (45.1)	3458 (55.1)	
Separated/Divorced	707 (7.3)	55 (1.6)	662 (10.4)	
Widowed	774 (7.9)	120 (3.5)	654 (10.4)	
**Economic activities**				
None	3484 (36.0)	1241 (36.5)	2243 (35.7)	<0.001
Farming/Livestock keeping	4675 (48.3)	1650 (48.5)	3025 (48.2)	
Skilled	1076 (11.1)	255 (7.5)	821 (13.1)	
Unskilled	443 (4.6)	255 (7.5)	188 (3.0)	
**Residence**				
Rural	6093 (62.9)	2345 (68.9)	3748 (59.7)	<0.001
Urban	3585 (37.0)	1056 (31.1)	2529 (40.3)	
**Ethnicity**				
Sukuma	8979 (92.8)	3201 (94.1)	5778 (92.1)	<0.001
Other	698 (7.2)	200 (5.9)	498 (7.9)	
		Mean (SD)		
BMI (kg/)	21.2 (3.6)	20.3 (2.8)	21.7 (3.9)	<0.001*
Systolic pressure (mmHg)	116.2 (15.7)	116.4 (14.5)	116.1 (16.4)	0.69*
Diastolic pressure (mmHg)	69.9 (12.4)	69.7 (12.9)	70.0 (12.1)	0.06*
Blood sugar (mg/dl)	100.1 (24.1)	99.1 (24.9)	100.6 (23.7)	<0.001*

All p-values are from chi-square test, except *t-test and **sum-rank test.

**Table 2.** Characteristics of the residents in Magu HDSS by blood pressure status (n = 9678).

Characteristics	Total	Normal n = 5398 (55.8%)	Pre-Hypertensive n = 3507 (36.2%)	Hypertensive n = 773 (8.0%)	p-value
**Sex**					
Male	3401	1883 (55.4)	1275 (37.5)	243 (7.7)	7.07
Female	6277	3515 (55.9)	2232 (35.6)	530 (8.5)	(0.029)
**Age (Years)**					
<24	3763	2490 (66.2)	1201 (31.9)	72 (1.9)	0.0011
25–34	2018	116 (57.6)	771 (38.2)	84 (4.2)	(<0.001)
35–44	1439	772 (53.7)	557 (38.7)	110 (7.6)	
45–54	1043	477 (45.7)	420 (40.3)	146 (14.0)	
55–65	618	225 (36.4)	252 (40.8)	141 (22.8)	
65+	704	224 (31.8)	263 (37.4)	217 (30.8)	
**Education level**					
None	2724	1324 (48.6)	1048 (38.5)	352 (12.9)	251.1
Not completed primary	658	307 (46.6)	273 (41.5)	78 (11.8)	(<0.001)
Completed primary	4798	2888 (60.2)	1641 (34.2)	269 (5.6)	
Secondary	1242	766 (61.7)	432 (34.8)	44 (3.5)	
Tertiary/Other	249	108 (43.4)	112 (45.0)	29 (11.6)	
**Marital status**					
Single/Never married	3200	2105 (65.8)	1012 (31.6)	83 (2.6)	613.6
Married/Cohabiting	4991	2639 (52.9)	1932 (38.7)	420 (8.4)	(<0.001)
Separated/Divorced	707	250 (35.4)	261 (36.9)	196 (27.8)	
Widowed	774	401 (51.8)	300 (38.8)	73 (9.4)	
**Occupation**					
None	3484	2143 (61.5)	1088 (31.2)	253 (7.3)	78.5
Farming/Livestock keeping	4675	2456 (52.5)	1825 (39.1)	394 (8.4)	(<0.001)
Skilled	1076	550 (51.1)	428 (39.8)	98 (9.1)	
Unskilled	443	249 (56.2)	166 (37.5)	28 (6.3)	
**Residence**					
Rural	6093	3361 (55.2)	2318 (38.1)	414 (6.8)	45.2
Urban	3585	2037 (56.8)	1189 (33.2)	359 (10.1)	(<0.001)
**BMI (kg/)**					
Underweight(18.5)	1915	1281 (66.9)	555 (28.9)	79 (4.1)	407.6
Normal (18.5–24.9)	6752	3734 (55.3)	2519 (37.3)	499 (7.4)	(<0.001)
Overweight (25–29.9)	758	318 (41.9)	322 (42.5)	118 (15.6)	
Obese (30)	239	57 (23.8)	108 (45.2)	74 (31.0)	
**Waist circumference**					
1st Tertile	3529	2128 (60.3)	1247 (35.3)	154 (4.4)	243.6
2nd Tertile	2986	1717 (57.5)	1082 (36.2)	187 (6.3)	(<0.001)
3rd Tertile	3153	1547(49.1)	1177 (37.3)	429 (13.6)	
**Alcohol consumption**					
Never	8817	4989 (56.6)	3163 (35.9)	666 (7.6)	38.0
Occasionally & Frequently	495	240 (48.5)	194 (39.2)	61 (12.3)	(<0.001)
**Cigarette smoking**					
Not currently	9035	5092 (56.3)	3243 (35.9)	700 (7.7)	22.7
Current	642	305 (47.5)	264 (41.1)	73 (11.4)	(<0.001)
**Physical activity**					
None	3794	2282 (60.2)	1231 (32.5)	281 (7.4)	48.9
Moderate /Heavy	5884	3116(52.9)	2276 (38.7)	492 (8.4)	(<0.001)
**Raised blood sugar**					
No (199.9 mg/dl)	9487	5306 (55.9)	3425 (36.1)	756 (8.0)	61.9
Yes (≥ 200 mg/dl)	27	4 (14.8)	10 (37.0)	13 (48.2)	(<0.001)
**HIV status**					
Negative	8940	4978 (55.7)	3228 (36.1)	734 (8.2)	8.0
Positive	738	420 (57.8)	279 (37.9)	39 (5.3)	(0.018)

Both hypertension and pre-hypertension increased with increasing levels of education. Participants living in rural areas had a higher prevalence of pre-hypertension (38.0%) and a lower prevalence of hypertension (6.8%) than those living in urban areas (33.2%, and 10.1% respectively). The prevalence for both pre-hypertension and hypertension increased with increase in body mass index (BMI), whereby those who were overweight (15.6%) and obese (31.4%) were more likely to have high blood pressure compared to participants with a BMI of 25 or less. A similar trend was observed for waist circumference in the largest tertile compared to those in the lowest tertile (Table 2). Participants who reported consumption of alcohol occasionally or frequently and those who were current cigarette smokers had slightly elevated blood pressure as compared to non-drinkers or non-smokers. The 27 participants with raised blood sugar levels had both higher proportions of pre-hypertension 37.0% and hypertension 48.2% compared with participants with normal blood sugar (36.1% and 8.0%) respectively.

## Bivariate and multivariate analysis

A number of demographic and clinical variables were significantly associated with increased odds (risk) of hypertension in bivariate analysis (Table 3). These included age, sex, marital status, area of residence, education level, waist circumference, BMI, alcohol consumption, and diabetes. There was no association between cigarette smoking and physical activities with the risk of hypertension.

**Table 3.** Univariate analysis for factors associated with hypertension in residence of Magu HDSS.

Characteristics	Total	Hypertensive n = 773 (8.0%)	OR (95% C.I)	p-value
**Sex**				
Male	3401	243 (7.1)	1.00 (Referent)	
Female	6277	530 (8.5)	1.19 (1.02 – 1.40)	0.025
**Age (Years)**				
15–24	3763	72 (1.9)	0.12 (0.09 – 0.16)	<0.001
25–34	2018	84 (4.1)	0.27 (0.20–0.35)	<0.001
35–44	1439	110 (7.6)	0.51 (0.39 – 0.66)	<0.001
45–54	1043	146 (14.0)	1.00 (Referent)	
55–64	618	141 (22.8)	1.82 (1.40 – 2.35)	<0.001
65+	704	217 (30.8)	2.74 (2.16 – 3.47)	<0.001
**Education level**				
None	2724	352 (12.9)	1.00 (Referent)	
Not completed primary	658	78 (11.8)	0.91 (0.69 – 1.78)	0.461*
Completed primary	4798	269 (5.6)	0.40 (0.34–0.47)	<0.001
Secondary	1242	44 (3.5)	0.25 (0.18–0.34)	<0.001
Tertiary/Other	249	29 (11.6)	0.89 (0.59–1.33)	0.565*
**Marital status**				
Single/Never married	3200	83 (2.6)	1.00 (Referent)	
Married/Cohabiting	4991	420 (8.4)	3.46 (2.71 – 4.39)	<0.001
Separated/Divorced	707	196 (27.8)	14.45 (10.99 – 18.99)	<0.001
Widowed	774	73 (9.4)	3.92 (2.83 – 5.42)	<0.001
**Residence**				
Rural	6093	414 (6.8)	1.00 (Referent)	
Urban	3585	359 (10.0)	1.53 (1.32 – 1.77)	<0.001
**Waist circumference**				
1^st^Tertile	3529	154 (4.4)	1.00 (Referent)	
2^nd^Tertile	2986	187 (6.3)	1.46 (1.18 – 1.82)	0.001
3^rd^Tertile	3153	429 (13.6)	3.45 (2.85 – 4.18)	<0.001
**BMI (kg/)**				
Underweight (≤18.5)	1915	79 (4.1)	0.54 (0.42 – 0.69)	<0.001
Normal (18.5–24)	6752	499 (7.4)	1.00 (Referent)	
Overweight (25–29)	758	118 (15.6)	2.31 (1.86 – 2.87)	<0.001
Obese (≥30)	239	74 (30.9)	5.62 (4.21 – 7.50)	<0.001
**Alcohol consumption**				
Never	8817	665 (7.5)	1.00 (Referent)	
Occasionally	496	62 (12.3)	1.75 (1.33 – 2.31)	<0.001
Frequent	364	46 (12.6)	1.77 (1.28 – 2.43)	<0.001
**Cigarette smoking**				
Not currently	9035	700 (7.8)	1.00 (Referent)	
Current	642	73 (11.4)	1.53 (1.18 – 1.97)	0.001
**Raised blood sugar**				
No (≤199.9 mg/dl)	9487	757 (7.9)	1.00 (Referent)	
Yes (≥ 200 mg/dl)	27	13 (48.2)	10.72 (5.02 – 22.89)	<0.001
**HIV status**				
Negative	8940	734 (8.2)	1.00 (Referent)	
Positive	738	39 (5.3)	0.62 (0.44 – 0.86)	0.005

In multivariate analysis after adjusting for all significant variables (P-value <0.1) from the bivariate analyses and the priori potential confounders; sex and age, waist circumference, BMI, blood sugar and HIV were found to be associated with hypertension (Table 4). Residence, occupation and alcohol consumption were not independently associated with the risk of being hypertensive.

**Table 4.** Multivariate logistic regression to determine the factors associated with hypertension in Magu HDSS.

Characteristics	Participants (n = 9678)	
	Adjusted OR (95% C.I)	p-value
**Sex**		
Male	1.00 (Referent)	
Female	0.64 (0.52 – 0.79)	<0.001
**Age(Years)**		
15–24	0.13 (0.09 – 0.20)	<0.001
25–34	0.27 (0.20–0.37)	<0.001
35–44	0.52 (0.39 – 0.68)	<0.001
45–54	1.00 (Referent)	
55–65	1.71 (1.30 – 2.26)	<0.001
65+	2.54 (1.90 – 3.39)	<0.001
**Waist circumference**		
1^st^ tertile	1.00 (Referent)	
2^nd^ tertile	1.07 (0.85 – 1.36)	0.551
3^rd^ tertile	1.36 (1.08 – 1.72)	0.008
**BMI(kg/)**		
Underweight (18.5)	0.51 (0.39 – 0.67)	<0.001
Normal (18.5–24.9)	1.00 (Referent)	
Overweight (25–29.9)	1.74 (1.35 – 2.25)	<0.001
Obese (30)	3.45 (2.44 – 4.86)	<0.001
**Raised blood sugar**		
No (≤199.9 mg/dl)	1.00 (Referent)	
Yes (≥ 200 mg/dl)	4.86 (2.06 – 11.48)	<0.001
**HIV Status**		
Negative	1.00 (Referent)	
Positive	0.56 (0.39 – 0.79)	0.001

Education level, marital status, residence, alcohol consumption and cigarette smoking were insignificant after adjustment.

Females had lower odds of beings hypertensive compared to males (OR = 0.69; 95% CI 0.56–0.85), while older people > 65 years had 2.5 higher odds of hypertension compared to middle-aged individuals (aged 45–54 years).Compared to single or never married women, separated or divorced women had 1.68 times the odds of being hypertensive even after adjusting for age. Cigarette smokers had 37% higher risk of being hypertensive compared to non-smokers.

Individuals living in urban areas were more at risk of being hypertensive (OR = 1.18,95% CI 0.99–1.41) compared to the ones in rural areas, although this association did not reach a statistical significant with P = 0.06, overweight and obese individuals had almost two to four times higher odds of being hypertensive compared to the ones with normal weight. There was a significant association between the 3rd tertile of waist circumference (OR = 1.39, 95% CI 1.11 – 1.76) and hypertension compared to the 1st tertile.

After adjusting for age-groups, education level had no significant association with hypertension as older people were more hypertensive and less educated. Occupation and alcohol consumption were not significantly associated with the risk of being hypertensive (P > 0.05), while people with HIV were found to have a 45% lower risk of raised blood pressure. Detailed results on the bivariate and multivariate analyses are given in Table 3 and Table 4.

Finally we assessed effect modification (interaction) by sex for each of the factors with hypertension, and found no significant interactions. For each sex, similar results were obtained to the combined multivariate analysis.

## Awareness and treatment of hypertension

**Figure 1.**
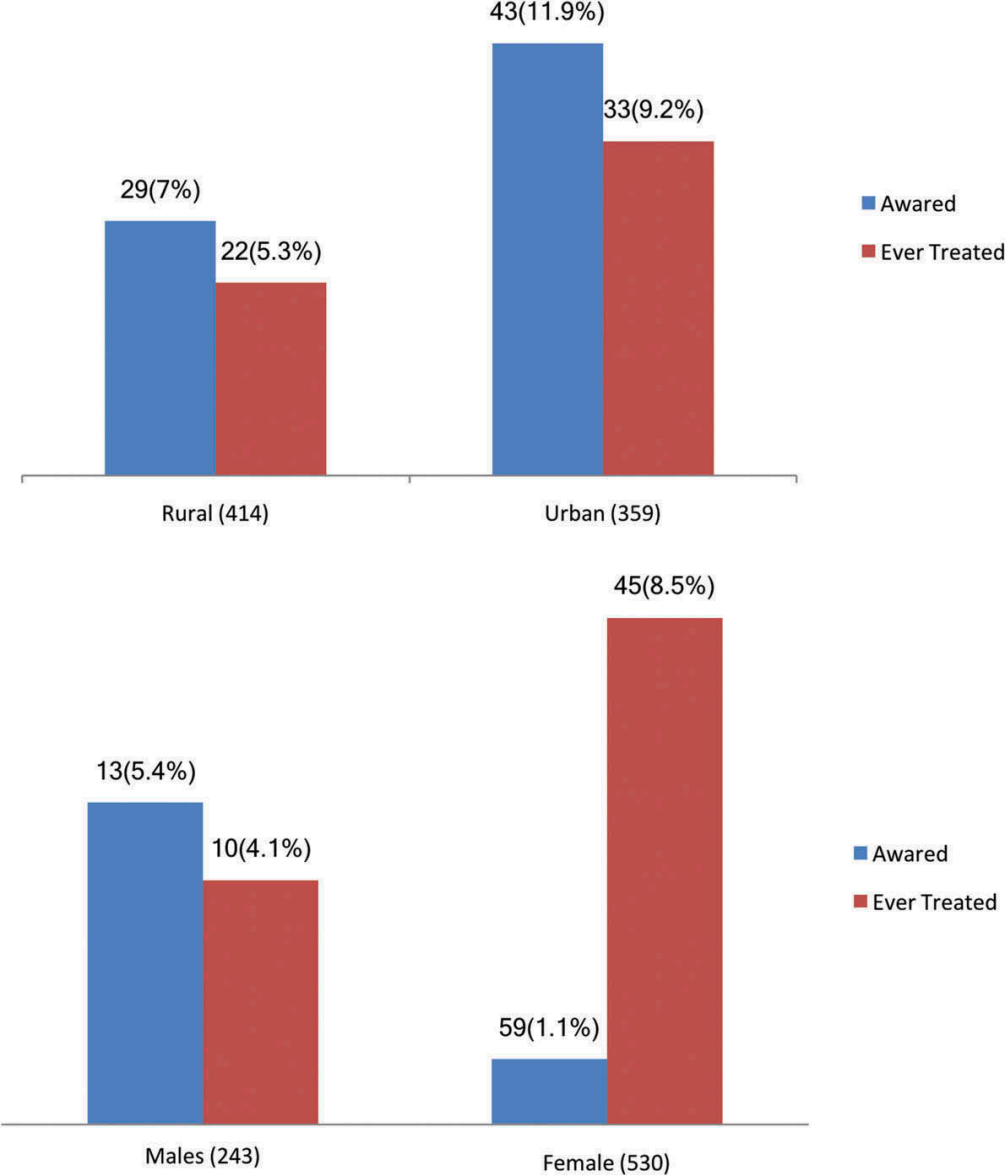
Awareness and treatment of hypertension by gender and residence among residents of Magu HDSS by sex (n = 773).

Figure 1 shows the results for awareness, and treatment of hypertension according to the gender of the 773 hypertensive participants. Of these, overall 72 (9.4%) participants were aware of their hypertension status, with significant differences between women (11.1%) and men (5.3%, P < 0.003), and between urban (11.9%) and rural dwellers (7%, p < 0.02)

Out of all 773 hypertensive individuals, 59 (7.1%) had been treated with antihypertensive drugs, with a higher frequency (9.2%) in urban areas compared to rural areas (5.3%, p < 0.03), and in females (8.5%) compared to males (4.1%, p < 0.03).

In all these aspects of hypertension management the elderly group aged 55 years and above were more aware, and more likely to be on treatment for hypertension for both male and female participants, although the differences were not statistically significant (p > 0.05).

## Discussion

After integrating blood pressure screening into a long-standing community HIV screening program, we identified 773 cases of hypertension, 8% of the screened population, despite the fact that the screened population was relatively young, with little obesity. The overall prevalence of 8.0% with raised blood pressure obtained from our study in a rural adult population was lower than the studies reviewed by Mfinanga [24] where the prevalence of hypertension was 14.5%, and lower than the study of individuals aged 18 years and above in Mwanza municipality by Kavishe [10] where the prevalence of hypertension was 16.4% (95% CI 11.7%-22.4%). However Mfinanga’s review included studies restricted to elderly individuals aged 70 years and above.

Despite having a lower prevalence of hypertension we had a high prevalence (35%) of pre-hypertension. Similar results have been found in a study in rural Tanzania where more than 45% of individuals aged 18 years and above, had pre-hypertension [4]. These findings highlight the need for urgent public health action to improve knowledge and awareness of hypertension in the population. Our study suggests that existing public health infrastructure such as the community HIV screening program described in this project, might provide valuable platforms for raising knowledge and awareness in the community.

One population-based study in Ethiopia found a prevalence of hypertension of 9.3%, which is similar to the prevalence in our study, although the study was restricted to participants aged 15–64 years. Other studies in Africa which have included individuals aged 15 years and above, have shown similar increases in the prevalence of hypertension across the age groups [6,25]. Our results show that age is associated with hypertension, with the prevalence rising to 30% in those aged 65 years or more, which agrees with other studies [2,26,27]. A few studies in Africa have explored hypertension by gender with one showing no difference after adjusting for age [28]. In our study we found a difference in the prevalence of hypertension between males and females after controlling for age and other risk factors.

This study found that the effect of alcohol consumption, HIV, and other risk factors on hypertension were similar in both sexes. The association between overweight, obesity, large waist circumference and hypertension was found in both males and females in our study. It is estimated that a 10 kg increase in body weight raises blood pressure by 3 mmHg systolic and 2.3mmHg diastolic blood pressure [16], and this association concurs with many other studies in Africa [2,26,27,29].Increased blood sugar was also associated with raised blood pressure, which also concurs with other studies worldwide [14].

However in our study alcohol consumption, cigarrete smoking, and physical activity were not independently associated with the risk of hypertension. These results differ from other studies in Africa where there was strong evidence of an association between these lifestyle factors and hypertension [15]. However our study had similar results to those obtained by Dzudie [25] in Cameroon, where after adjusting for other socio-demographic factors no association was seen between hypertension and these lifestyle factors. The lack of association in our study might be due to the categorization of alcohol and smoking usage, but is also affected by the small numbers engaging in these risk behaviours in this predominately rural population.

There was a negative association between HIV-positive infected participants and the risk of hypertension, which agrees with findings by Peck in patients that are antiretroviral therapy (ART) naïve [30].The association between HIV and hypertension has caused many public health experts to argue for the integration of HIV and hypertension services in Africa. Integration of these services could have the benefits of extending the life span of HIV-infected adults and of increasing ART adherence. Possible risks, though include the financial costs of providing hypertension care in an HIV clinic as well as the human resource issues if health care providers in these clinics are asked to do more work.

Despite hypertension emerging as a public health problem in Sub-Saharan Africa, and being a common cause for cardiovascular disease, the rates of hypertension treatment are still very low. Even in developed countries, studies have shown that the awareness and management of hypertension are low, especially in men [27,31]. Our study shows similar findings with <10% of participants with hypertension reporting awareness of their status and only 7% reporting previously receiving treatment. These rates of awareness are even lower than other studies from Africa which showed awareness rates of 29% [32] and 18% [2] . A study in Ghana found similar results to our study with 7.4% of those with hypertension aware of their condition, 4.0% on treatment, and 3.5% having controlled blood pressure [33]. The low rates of treatment are likely related to problems with cost and availability of hypertension drugs. In our study setting, for example, hypertension drugs are often not available in public clinics. Although hypertension drugs are available in private clinics and pharmacies, the monthly cost of these drugs often is greater than the average monthly income of many families.

These results shows that there is an urgent need for interventions to raise awareness of hypertension, and to help modify lifestyle behaviors in reducing the risk of raised blood pressure. This can include information on the use of alcohol and smoking as well as encouragement to maintain a good diet, as this population is mostly rural, with farming being the main occupation, and located just 20 km from the second largest city in Tanzania. Management of the demographic transition in this population must include advice about lifestyle and dietary choices to reduce the risk of hypertension and to control their blood pressure.

## Conclusion

The findings from our study show that hypertension is high even in rural Tanzania, especially in older people. The pre-hypertension prevalence of 36.2% means that more individuals are at risk of developing hypertension, which is a major concern to health planners. Modification of lifestyle through knowledge provision and dietary intake will be an effective strategy in hypertension management, but this requires a strong, national public health messages. Through the integration of the screening for hypertension into routine clinics for HIV, diabetes, and other chronic diseases, many undiagnosed cases can be identified. If this is combined with proper treatment of hypertension then the consequences, such as cardiovascular disease and stroke, can be avoided, but this requires improvements of the clinical services in health facilities.

## Paper context

Hypertension is a public health problem; and as Tanzania is now facing nutrition transition the risk of non-communicable diseases increases. Hence, appropriate measures should be taken by identifying and managing these patients. Determining the magnitude, awareness, and the contributing factors of hypertension in this HIV-surveillance community will be an added advantage. Findings from this study will have an important role in provision of preliminary information on the magnitude and severity of the problem and will also help in identifying proper management strategies as now we are faced with a double burden of diseases.
